# A Google Trends Analysis of Search Interest for Tender-Headedness and Scalp-Related Concerns

**DOI:** 10.2196/60040

**Published:** 2025-02-13

**Authors:** Charissa Obeng-Nyarko, Tatiana Barrera, Temitayo Ogunleye, Susan Taylor

**Affiliations:** 1 Florida State University College of Medicine, 1115 West Call Street, Tallahassee, FL, 32306, United States, 1 850 644 1855; 2 University of California - Riverside, Riverside, CA, United States; 3 Department of Dermatology, University of Pennsylvania, Philadelphia, PA, United States

**Keywords:** tender-headedness, tender-headed, scalp tenderness, dermatologists, Google Trends, Black patients

## Abstract

In this Google Trends cross-sectional analysis, we aimed to understand the popularity of tender-headedness by analyzing related Google search queries from January 2013 to December 2022. Since 2013, Google searches on scalp-related concerns, especially those regarding tender-headedness in Black hair culture, have increased, thus uncovering an opportunity for dermatologists to utilize culturally relevant language to address scalp concerns in patients with Afro-textured hair.

## Introduction

In Black hair culture, “tender-headed” is a term that refers to someone with heightened scalp discomfort or tenderness during hair manipulation procedures like combing, brushing, braiding, twisting, hair parting, and blow-drying [1-3].

Little is known about tender-headedness, as it usually lacks clinical findings. However, symptoms include mild to significant scalp discomfort, which can occur among all ethnicities but may be more prevalent among women with Afro-textured hair [4]. Scalp tenderness is a common symptom in inflammatory alopecias, including central centrifugal cicatricial alopecia and traction alopecia, which predominantly affect Black women [5]. Understanding culturally relevant language for scalp tenderness is important for dermatologists to differentiate between nonpathologic and pathologic scalp issues in this population.

The internet is a commonly used source for information on hair and scalp care, particularly for people of African descent who may seek solutions for tender-headedness on search engines and forums [6]. To date, there is limited knowledge about internet search interest regarding tender-headedness. In this study, we aim to understand the popularity of tender-headedness by analyzing search queries related to this concept on a major search engine.

## Methods

Google Trends (GT) is a Google-developed tool that reports on the popularity of specific searches. Output from GT is in the form of a search volume index (SVI), which represents the popularity of a specific search over time [7]. SVI values are normalized on a scale from 0 to 100, with 0 representing the lowest level of interest and 100 representing the highest [7]. These values depend on the specific search phrase, time range, and geographical area selected [7]. They may vary slightly by query date, so all values were queried on the same day for consistency [7].

In this cross-sectional analysis, GT was used to extract the monthly web SVI from January 2013 to December 2022 for the following seven keyword phrases (KPs): “tender headed,” “tender head,” “sore scalp,” “scalp hurts,” “tight scalp,” “tender scalp,” and “scalp tenderness.” Additionally, KPs were grouped into the following three categories of generic words used to describe tender-headedness: (1) tenderness (“tender headed” and “tender head”), (2) scalp discomfort (“sore scalp,” “scalp hurts,” and “tight scalp”), and (3) both concepts (“tenderness” and “scalp”) combined (“tender scalp” and “scalp tenderness”). Differences in the mean monthly SVI per category were compared via a generalized estimated equation with Gaussian estimation and exchangeable correlation, using Stata version 18 (StataCorp LLC). Statistical significance was measured at *P*<.05.

## Results

**Figure 1.**
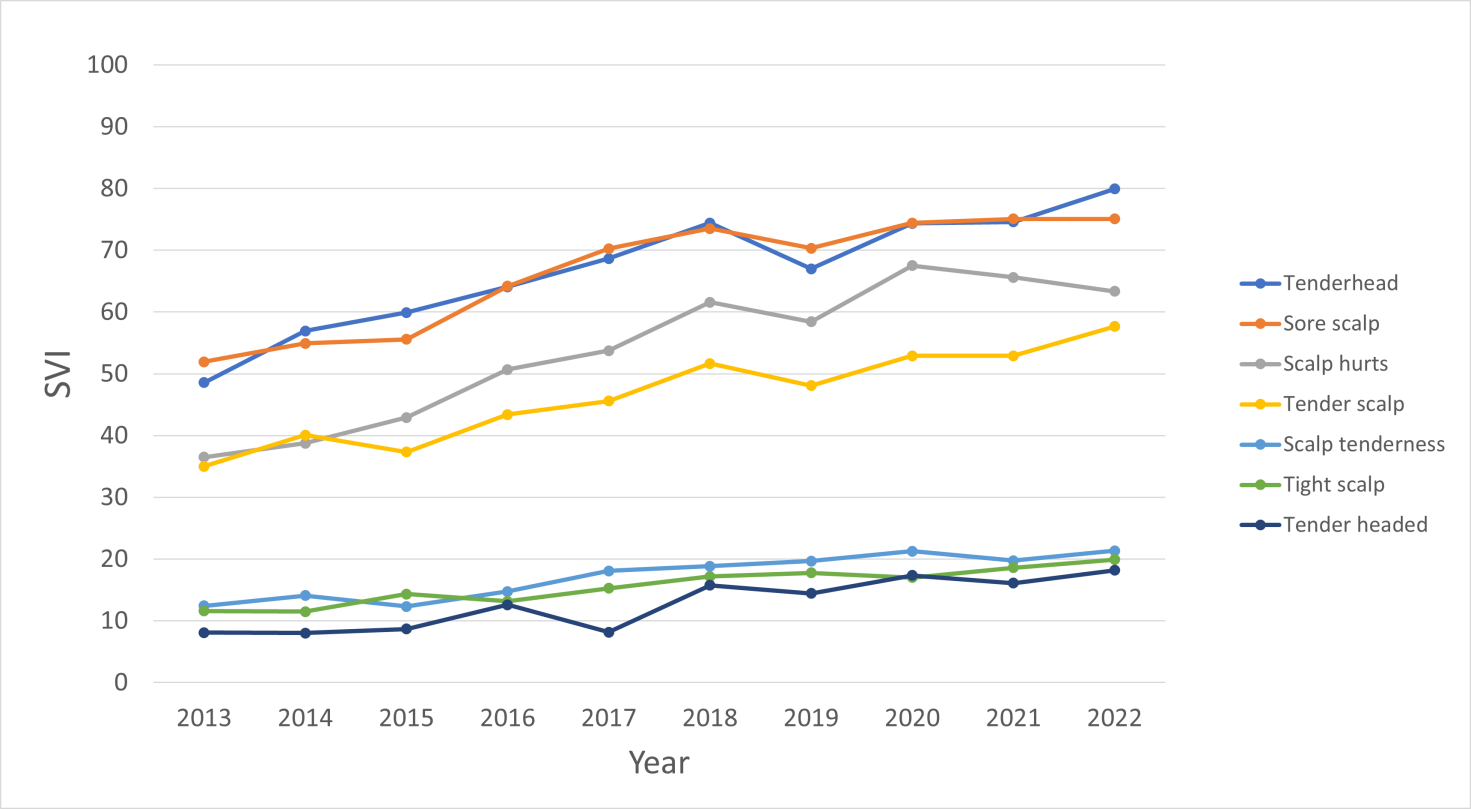
Yearly internet search interest by keyword phrase from 2013 to 2022. SVI: search volume index.

Among the seven KPs used in US internet queries made between January 2013 and December 2022, “tender head” and “sore scalp” had the highest mean SVIs (67 for both; Figure 1). The internet search interest for the term “sore scalp” was comparable to that for “tender head” (*R*=0.32, 95% CI −1.23 to 1.87; *P*=.69). The term “tender headed” yielded lower search interest compared to “tender head” (*R*=−53.3, 95% CI −55.08427 to −51.98239; *P*<.001).

**Figure 2.**
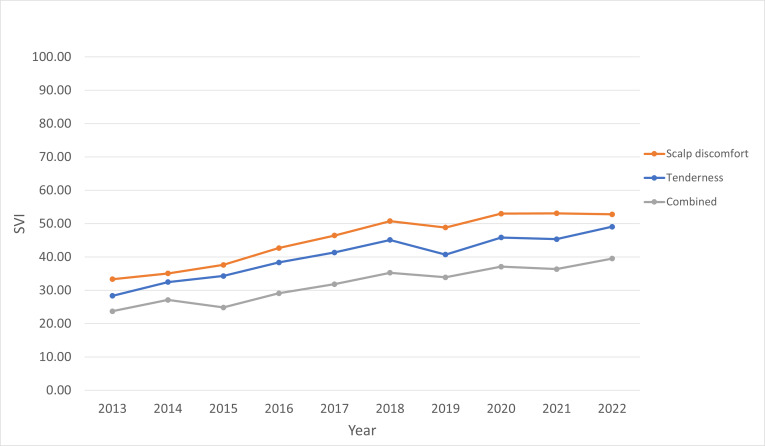
Yearly internet search interest by keyword phrase category from 2013 to 2022. SVI: search volume index.

The “scalp discomfort” category had the highest mean internet search interest (SVI=45.35) compared to the “tenderness” (SVI=40.08) and “combined” (SVI=31.86) categories (Figure 2). When compared to searches for the “tenderness” category, there was significantly higher search interest for the “scalp discomfort” category (*R*=5.07, 95% CI 4.21-5.93; *P*<.001). Additionally, the “combined” category showed significantly lower interest in comparison to “tenderness” (*R*=−8.19, 95% CI −9.05 to −7.33; *P*<.001).

## Discussion

In this study, we found that terms describing scalp discomfort generated the highest internet search interest among KP categories. Furthermore, among the seven KPs describing tender-headedness, “tender head” and “sore scalp” had the greatest internet search volume.

Although our findings did not show a solid search trend for the keyword “tender headed,” “tender head” and “sore scalp” are relevant phrases that some individuals use to describe tender-headedness. “Tender head” has gained popularity on the web, with search engine queries yielding culturally specific articles on managing tender-headedness in children and adults with Afro-textured hair [2,3,8,9] and the need for dermatologic care [3,8,9]. Understanding this context may benefit dermatologists when discussing scalp concerns with Black patients.

This study acknowledges that using GT to capture internet search interest for scalp concerns may not fully represent individuals without internet access, limiting the results’ applicability to the broader population [7]. Despite this limitation, GT is a powerful tool for gauging public interest in dermatology-related terms and conditions [7].

Since 2013, Google searches on scalp-related concerns, especially those regarding tender-headedness in Black hair culture, have increased. Further research is needed to characterize tender-headedness and understand its relationship with hair and scalp disorders in people of African descent.

## Abbreviations

GT: Google Trends
KP: keyword phrase
SVI: search volume index
